# An interpretable RNA foundation model for exploring functional RNA motifs in plants

**DOI:** 10.1038/s42256-024-00946-z

**Published:** 2024-12-09

**Authors:** Haopeng Yu, Heng Yang, Wenqing Sun, Zongyun Yan, Xiaofei Yang, Huakun Zhang, Yiliang Ding, Ke Li

**Affiliations:** 1 Key Laboratory of Molecular Epigenetics of the Ministry of Education, Northeast Normal University, Changchun, China; 2 Department of Cell and Developmental Biology, John Innes Centre, Norwich Research Park, Norwich, UK; 3 Department of Computer Science, University of Exeter, Exeter, UK; 4 State Key Laboratory of Plant Genomics and National Center for Plant Gene Research (Beijing), Institute of Genetics and Developmental Biology, Innovation Academy for Seed Design, Chinese Academy of Sciences, Beijing, China; 5 National Key Laboratory of Plant Molecular Genetics, CAS Center for Excellence in Molecular Plant Sciences, Institute of Plant Physiology and Ecology, Chinese Academy of Sciences, Shanghai, China; 6 CAS-JIC Center of Excellence for Plant and Microbial Sciences, Institute of Plant Physiology and Ecology, Chinese Academy of Sciences, Shanghai, China; 7 Alan Turing Institute, London, UK

**Keywords:** Computational models, Machine learning, Plant molecular biology

## Abstract

The complex ‘language’ of plant RNA encodes a vast array of biological regulatory elements that orchestrate crucial aspects of plant growth, development and adaptation to environmental stresses. Recent advancements in foundation models (FMs) have demonstrated their unprecedented potential to decipher complex ‘language’ in biology. In this study, we introduced PlantRNA-FM, a high-performance and interpretable RNA FM specifically designed for plants. PlantRNA-FM was pretrained on an extensive dataset, integrating RNA sequences and RNA structure information from 1,124 distinct plant species. PlantRNA-FM exhibits superior performance in plant-specific downstream tasks. PlantRNA-FM achieves an F1 score of 0.974 for genic region annotation, whereas the current best-performing model achieves 0.639. Our PlantRNA-FM is empowered by our interpretable framework that facilitates the identification of biologically functional RNA sequence and structure motifs, including both RNA secondary and tertiary structure motifs across transcriptomes. Through experimental validations, we revealed translation-associated RNA motifs in plants. Our PlantRNA-FM also highlighted the importance of the position information of these functional RNA motifs in genic regions. Taken together, our PlantRNA-FM facilitates the exploration of functional RNA motifs across the complexity of transcriptomes, empowering plant scientists with capabilities for programming RNA codes in plants.

## Abstract (web summary)

Approaches are needed to explore regulatory RNA motifs in plants. An interpretable RNA foundation model is developed, trained on thousands of plant transcriptomes, which achieves superior performance in plant RNA biology tasks and enables the discovery of functional RNA sequence and structure motifs across transcriptomes.

## Main

The transcriptome contains a wide array of RNA motifs that impact diverse biological functions such as translation^1–5^. These RNA motifs encompass both RNA sequence and structure features. Previous individual studies have revealed the functional importance of RNA sequence features such as the Kozak sequence motif^6^. Recently, our studies along with others have suggested that both RNA secondary and tertiary structure motifs play important roles in diverse biological processes^7–13^. Particularly in plants, the relatively low habitat temperatures (~20 °C) favour the folding of RNA structure motifs, including RNA tertiary motifs such as RNA G-quadruplexes (rG4s)^12^. However, systematically identifying functional RNA motifs across transcriptomes remains a formidable challenge due to the high level of complexity arising from astronomical combinations of the four nucleotide bases into tens of thousands of transcripts^8,14^. For example, for a 50-nucleotide sequence, the number of artificially synthesized sequences would be on the order of 4^50^ (approximately 1.27 × 10^30^), which is impossible to achieve experimentally. Additionally, the functional readouts using the reporter gene assay for measuring biological functions such as translation may not be sensitive enough to detect differences in individual single-nucleotide mutations^15^.

The recent proliferation of foundation models (FMs) in artificial intelligence (AI) are set to show exciting promise for supercharging scientific advances in life sciences^16^. FMs are distinguished by their massive scale, often encompassing millions to billions of parameters. They are pretrained in a self-supervised manner on diverse forms of unlabelled data. This makes them ideal for bioscience, where acquiring abundant labelled data is prohibitively expensive and time-consuming. More importantly, FMs are highly adaptable through fine tuning and are poised to aid bioscientists in customizing generalist FMs for unravelling complex biological processes, paving the way for unprecedented capabilities in modulating gene functions. For FMs on DNA sequences, DNABERT-2 is one of the FMs pretrained on the genome sequences across 135 species, including mammals, fungi and bacteria^17^. By pretraining on diverse human and non-human genomes, the nucleotide transformer (NT) family learns transferable representations that enable accurate molecular phenotype prediction with limited annotated data and focus on key genomic elements without supervision^18^. FMs have also achieved success in protein sequences, also known as protein language models. For example, evolutionary-scale modelling (ESM2) has achieved remarkable breakthroughs in atomic-level structure representations by pretraining on a vast amount of protein sequences and structures^19^.

**Fig. 1:**
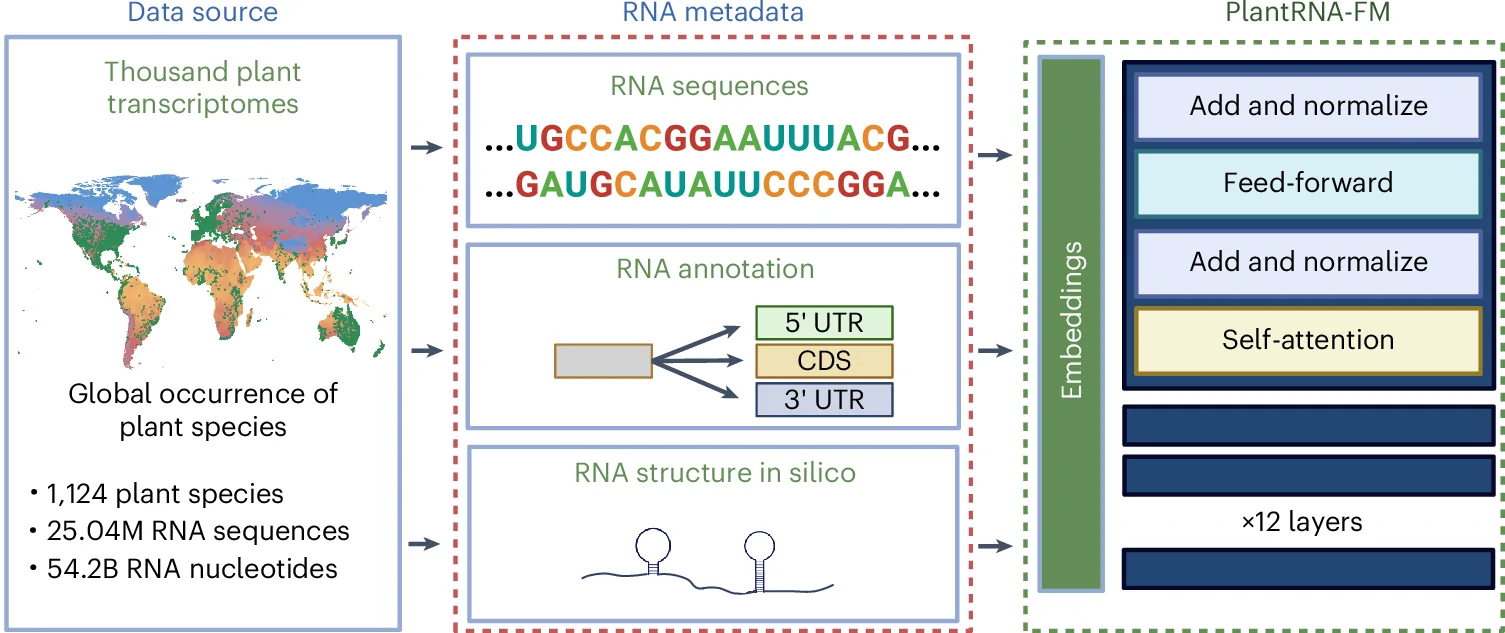
Schematic of the pretraining phase of PlantRNA-FM. The pretraining dataset comprises transcriptomic sequences from 1,124 plant species, consisting of approximately 25.0M RNA sequences and 54.2B RNA bases. The green dots on the global mean temperature map represent the geographical distribution of these plant species across the world. Basemap from (https://www.naturalearthdata.com). Figure created with BioRender.com.

For understanding RNA, several FMs were pretrained using RNA sequence information that has demonstrated great performance in RNA molecule design^20–23^. However, RNA sequence information is insufficient since RNA can form secondary or tertiary structure motifs that are important for its functions^24,25^. Therefore, it is important to generate an RNA FM including both RNA sequence and structure information to facilitate the exploration of functional RNA motifs. Here we developed PlantRNA-FM, a groundbreaking RNA FM designed to globally identify functional RNA motifs, including both RNA sequences and structure motifs in plants (Fig. 1). By incorporating RNA sequences, annotations and structure information from 1,124 distinct plant species, PlantRNA-FM captures the extensive diversity of plant transcriptomes (Fig. 1). We validate the superior performance of PlantRNA-FM in downstream tasks compared with existing FMs. Furthermore, we also established an interpretable framework based on our PlantRNA-FM to determine the critical regions across the 5′ untranslated regions (5′ UTRs) that significantly impact translation. Remarkably, PlantRNA-FM identifies RNA motifs at the transcriptome-wide scale that are functionally important to translation including both RNA sequences and secondary and tertiary structure motifs. We further experimentally validated these identified RNA motifs in plants. The development of our PlantRNA-FM represents a notable leap forward in our ability to decipher hidden regulatory codes among the extensive complexity of nucleotides across the transcriptome, opening new avenues for RNA-based gene regulation.

## Results

### PlantRNA-FM integrates 1,124 plant transcriptomes

The plant kingdom encompasses approximately 500,000 species, exhibiting remarkable diversity. The One Thousand Plant Transcriptomes Initiative (1KP) sequenced the transcriptomes of 1,124 species, capturing the extensive diversity of plant transcriptomes^14^. Here we took advantage of this unique resource and generated the pretraining dataset for our PlantRNA-FM (Fig. 1). Different from existing FMs, our PlantRNA-FM was designed to capture and learn both RNA sequences and RNA structure motifs. We used RNAfold^26^ to predict the RNA structures of individual RNA sequences across 1,124 transcriptomes and integrated them into the pretraining dataset. Our PlantRNA-FM has 35 million parameters, including 12 transformer network layers, 24 attention heads and an embedding dimension of 480 been optimized for RNA understanding rather than generation (Methods). Our tokenization approach ensures the preservation of RNA structure motifs as coherent units throughout the pretraining process (Methods). In addition, we incorporated RNA annotation information (CDS and UTRs) and used advanced pretraining techniques, such as sequence truncation, filtering and masked nucleotide modelling (MNM) (Methods).

**Extended data Fig. 1:**
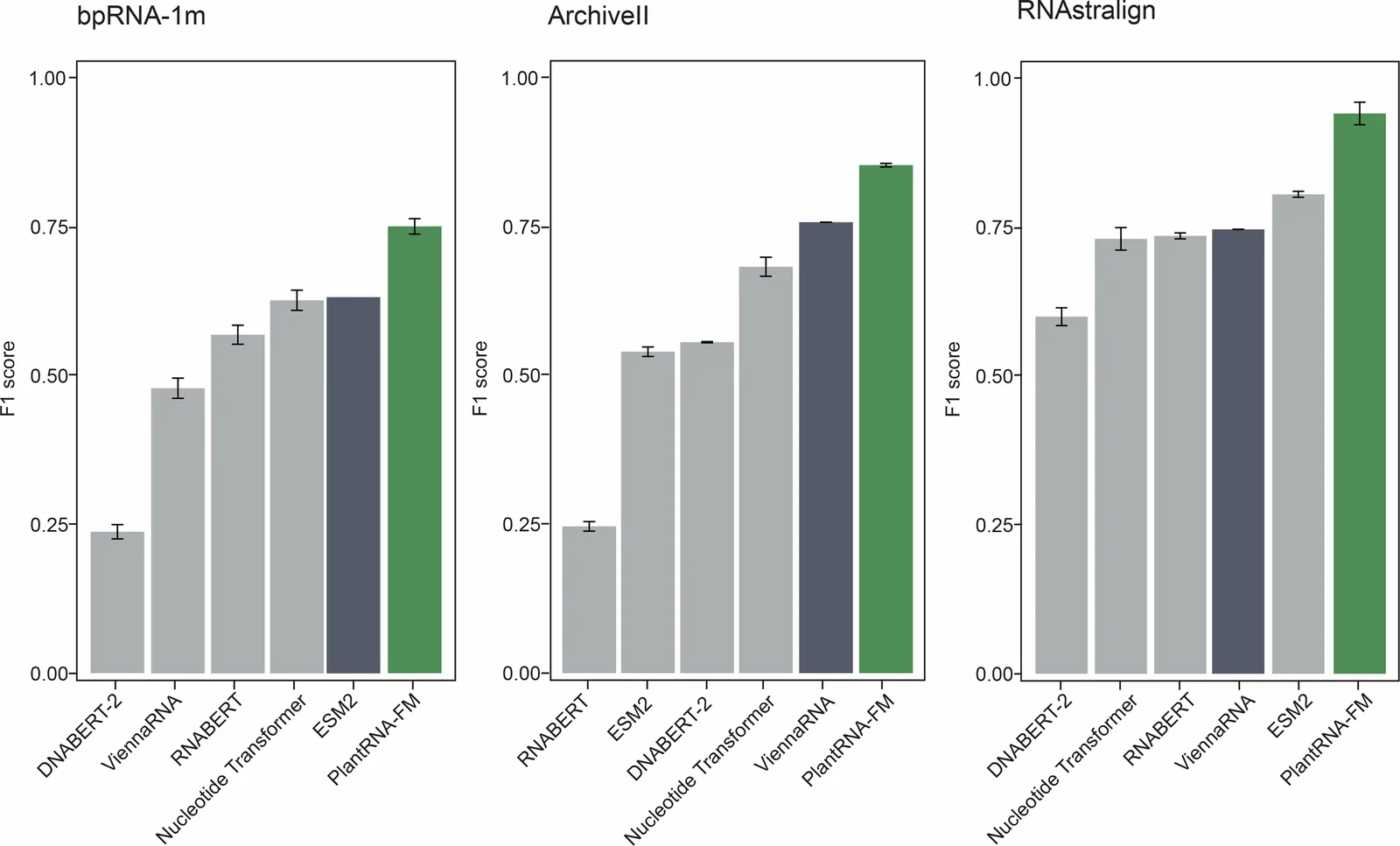
Comparison of the model performance of different pre-trained models on the RNA secondary structure prediction task. Three classic RNA secondary structure datasets were used to evaluate the performance of different pre-trained models. The most widely used ViennaFold software was also considered and marked in dark colour. PlantRNA-FM is highlighted in green. Error bars indicate SD. Source data

To assess the effectiveness of our PlantRNA-FM in RNA structure prediction tasks, we evaluated its performance (Extended Data Fig. 1 and Supplementary Table 1) using three benchmark datasets: bpRNA (TR0-TS0), ArchiveII and RNAStralign^27–29^. The F1 score, which is the harmonic mean of precision and recall, was used to measure the model’s predictive performance on these datasets. The F1 scores achieved by our PlantRNA-FM on these three datasets were 0.750, 0.924 and 0.981, respectively, whereas RNAfold alone only obtained F1 scores of 0.278, 0.759 and 0.748 (Extended Data Fig. 1 and Supplementary Table 1). Compared with other state-of-the-art FMs, PlantRNA-FM outperformed the second-best model by 0.124, 0.171 and 0.136 on the respective datasets (Extended Data Fig. 1 and Supplementary Table 1). Therefore, the unique integration of RNA structure information equips our PlantRNA-FM with the ability to predict RNA structure more accurately.

### PlantRNA-FM reveals superior performance on downstream tasks

**Fig. 2:**
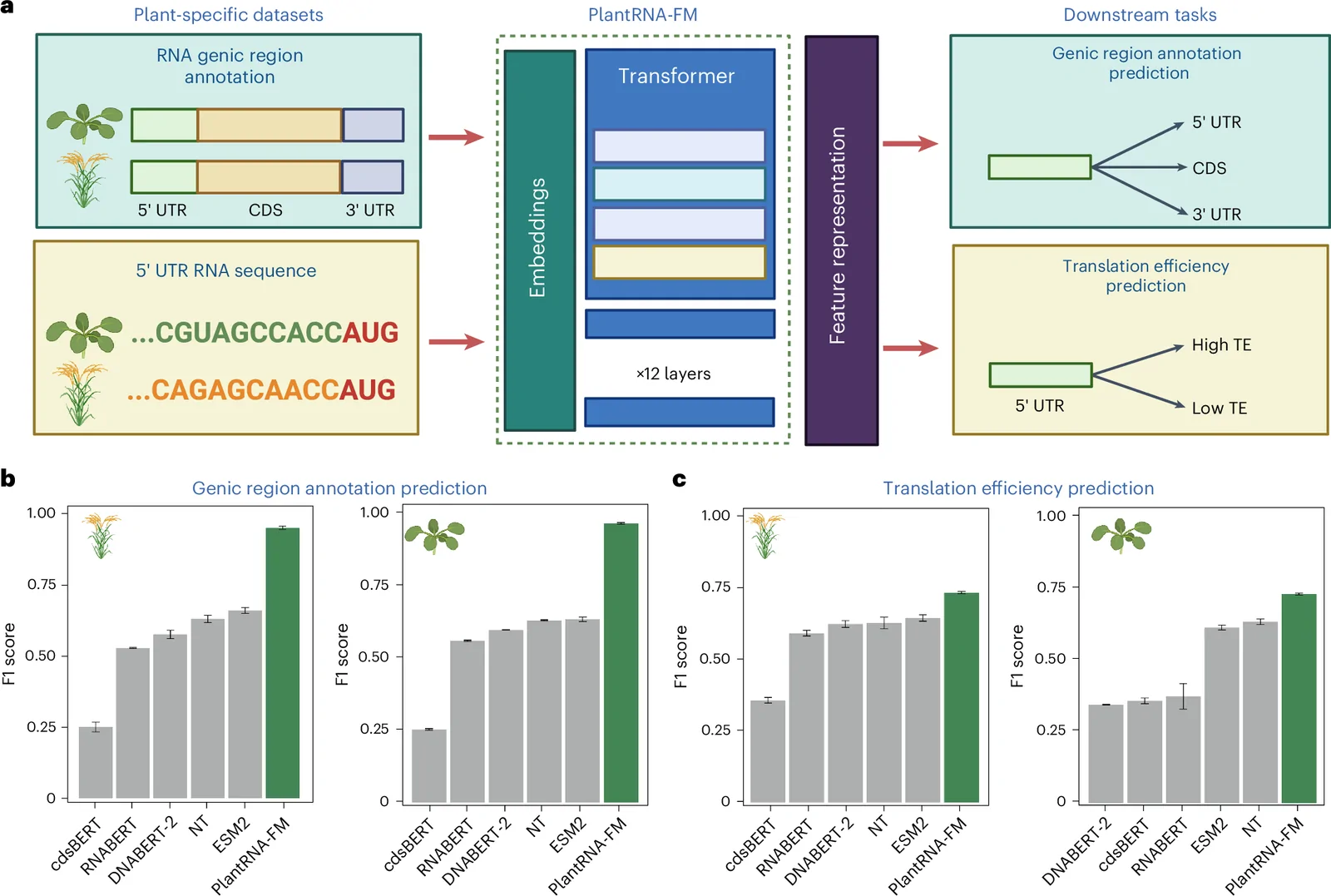
Fine-tuning PlantRNA-FM on plant-specific datasets. **a**, Overview of fine tuning PlantRNA-FM for RNA genic region annotation prediction and RNA TE prediction tasks. *A. thaliana* and *O. sativa* were selected as the representative plant species. For the RNA genic region annotation prediction task, RNA sequences from these two species were included, along with three labels: 5′ UTR, CDS and 3′ UTR. For the RNA TE prediction task, the 5′ UTR sequences from these two species were included, along with TE labels (high TE and low TE). **b**,**c**, Comparison of the model performance of different pretrained models on RNA genic region annotation prediction and RNA TE prediction tasks. The error bars represent the standard deviation of the F1 scores obtained from three fine-tuning replicates. Panel **a** was created with BioRender.com. Source data

To evaluate the performance of PlantRNA-FM, we curated a benchmark set consisting of four other state-of-the-art FMs: DNABERT-2, RNABERT, NT, ESM2 and cdsBERT^17–19,21,23^. We assessed their performance in two plant-specific downstream tasks: genic region annotation and translation efficiency (TE) prediction (Fig. 2a).

In the RNA genic region annotation prediction task, we aimed to identify and classify different genic regions of given RNA sequences, such as the 5′ UTR, coding sequence (CDS) and 3′ UTR. We used the transcriptomes of two model plant species, namely, *Arabidopsis thaliana* (a dicot model plant) and *Oryza sativa* L. ssp*. japonica* (rice, a moncot model plant). Both of them were not included in our pretraining dataset. For the RNA genic region annotation prediction in these two species, our PlantRNA-FM outperformed other FM models, achieving average F1 scores of 0.974 and 0.958 for *Arabidopsis* and rice, respectively, surpassing the second-best model by 0.339 and 0.293 (Fig. 2b and Table 1).

**Table 1:** Comparison of F1 scores achieved by different pretrained models on benchmark datasets

Tasks	Species	PlantRNA-FM	RNABERT	cdsBERT	DNABERT-2	NT	ESM2
RNA genic region annotation prediction	*A. thaliana*	0.974 ± 0.003	0.564 ± 0.002	0.254 ± 0.003	0.602 ± 0.001	0.635 ± 0.002	0.639 ± 0.008
	*O. sativa*	0.958 ± 0.006	0.532 ± 0.002	0.252 ± 0.017	0.580 ± 0.015	0.635 ± 0.013	0.665 ± 0.010
RNA TE prediction	*A. thaliana*	0.735 ± 0.003	0.375 ± 0.045	0.359 ± 0.010	0.346 ± 0.001	0.637 ± 0.010	0.617 ± 0.008
	*O. sativa*	0.737 ± 0.004	0.595 ± 0.01	0.359 ± 0.010	0.627 ± 0.012	0.631 ± 0.020	0.649 ± 0.011

For translation, in one of the key RNA biological processes, previous research has highlighted the critical role of the 5′ UTR in regulating translation efficiencies^17–19,21,30–32^. To evaluate the TE prediction performance of our PlantRNA-FM, we used the 5′ UTR sequences of both *Arabidopsis* and rice transcriptomes along with the corresponding TE values measured by polysome profiling^8^. We first classified the TE datasets into high and low TE groups, using the mean ± standard deviation as the threshold. In the TE prediction task, PlantRNA-FM achieved F1 scores of 0.735 and 0.737 for *Arabidopsis* and rice, respectively, outperforming the second-best model (Fig. 2c). Taken together, our PlantRNA-FM is better suited for plant-specific downstream tasks compared with other FMs pretrained on non-plant datasets.

### Interpretable PlantRNA-FM revealed important RNA features

**Fig. 3:**
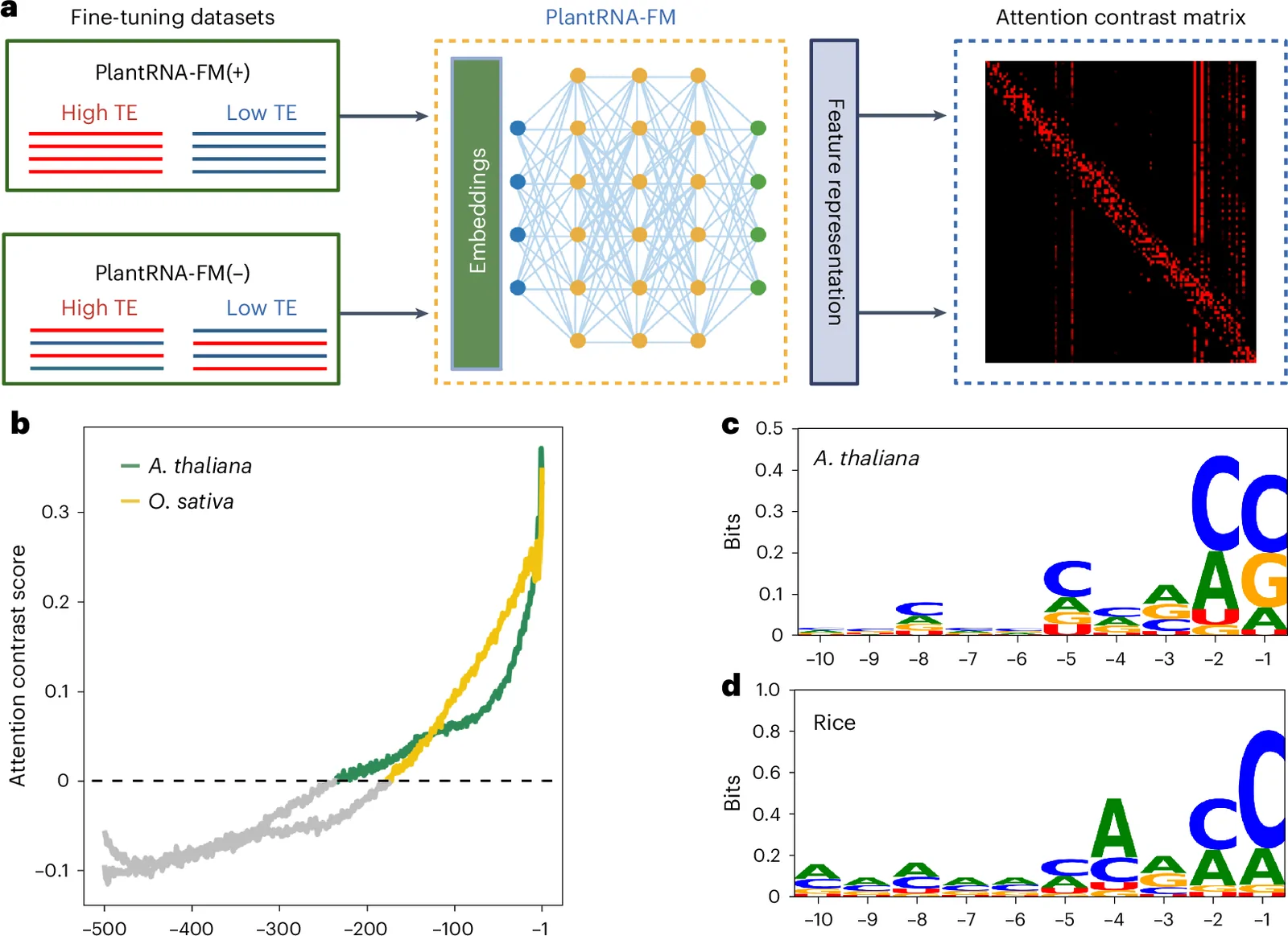
Model interpretable framework reveals translation-associated RNA features. **a**, Schematic of the model interpretability approach. **b**, Transcriptome-wide attention contrast scores. The –1 position represents the first site upstream of the AUG. Different species are distinguished by colours. **c**,**d**, Information content of the ten high attention bases closest to the AUG start codon. Panel **a** was created with BioRender.com. Source data

A general roadblock in applying AI models to biology is that although these models demonstrate strong predictive capabilities, the key to their successful application lies in interpreting them to uncover the biological principles learned by AI. In this paper, we established an interpretable framework to derive an attention contrast matrix from our PlantRNA-FM (Methods). In particular, we are interested in extracting the key RNA features within the 5′ UTR that significantly impact RNA translation, that is, elucidating the RNA motifs associated with translation (Fig. 3a). We developed two models in parallel: one is the true model, denoted as PlantRNA-FM(+), trained using the real TE dataset, whereas the other one is called the background model, PlantRNA-FM(–), altered using the same dataset but with randomly assigned labels (Fig. 3a). The F1 score achieved by the background model is approximately 50%, which is close to the random chance (mean F1 = 0.522), whereas the true model attained a substantially higher mean F1 score of 0.737. This indicates that PlantRNA-FM(+) has successfully learned the RNA features in the 5′ UTR sequences associated with translation.

By subtracting the attention matrices of the background model from those of the true model, we obtained an attention contrast matrix that highlighted the importance of nucleotides in the 5′ UTR contributing to TE (Fig. 3a). Across the transcriptomes, we observed an increase in attention contrast scores as the position approached the AUG start codon in both *Arabidopsis* and rice (Fig. 3b). This result indicates that positions close to the start codon contribute most to the TE values. By underlining the RNA sequence contents with a high-contrast attention score (identified by a *z* score of >2.326), our PlantRNA-FM successfully identified the Kozak sequence motifs in both *Arabidopsis* and rice transcriptomes that are associated with TE (Fig. 3c,d). This result demonstrates that our PlantRNA-FM successfully identifies evolutionarily conserved RNA motifs that are important to translation (Fig. 3c,d).

### PlantRNA-FM identifies the functional RNA structure motifs

**Fig. 4:**
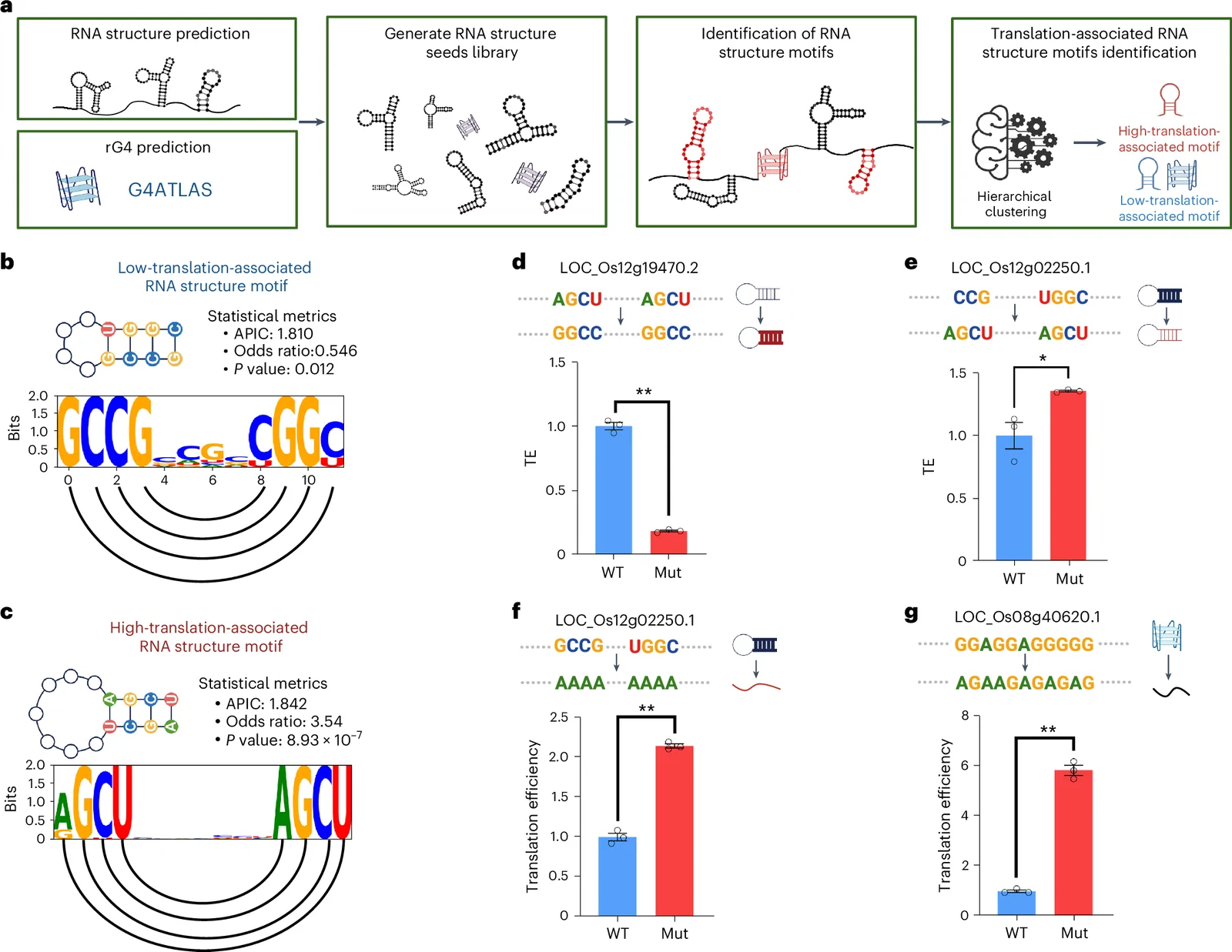
RNA structure motif identification approach reveals translation-associated RNA structure motifs. **a**, Overview of the RNA structure motif identification approach. RNA structures are predicted using RNAfold with a maximum length of 30 nucleotides to obtain the RNA structure seeds. Predicted rG4s were obtained from the G4Atlas database. **b**,**c**, Schematic of high-translation-associated RNA structure motifs and low-translation-associated RNA structure motifs. The sequence logos show the information content of each nucleotide, with semicircles connecting the paired bases. The *P* value is derived from a two-sided Fisher’s exact test. APIC, average positional information content. **d**–**g**, Experimental validation of high- and low-translation-associated RNA structure motifs and low-translation-associated rG4. The bar plot represents the translational efficiency of the original (WT) and RNA-structure-mutated (Mut) constructs from the dual-luciferase reporter assay in plants. It represents the change from high-translation-associated RNA structure motifs to low-translation-associated RNA structure motifs (**d**), the change from low-translation-associated RNA structure motifs to high-translation-associated RNA structure motifs (**e**), the complete disruption of low-translation-associated RNA structure motifs (**f**) and the complete disruption of low-translation-associated rG4 (**g**). *P* values (**d**–**g**) are 9.677 × 10^–^^6^, 0.027, 2.897 × 10^–5^ and 2.004 × 10^–5^, respectively, obtained using a two-sided Student’s *t*-test. * indicates *P* < 0.05, ** indicates *P* < 0.01, *n* = 3; error bars indicate the standard error. Panel **a** was created with BioRender.com. Source data

Since RNA structure is the unique RNA feature incorporated in our PlantRNA-FM, we further identified the RNA secondary structure motifs important to translation through the model’s attention contrast matrix and an unsupervised hierarchical clustering strategy (Fig. 4a and Methods). Overall, we identified 112 RNA secondary structure motifs that are important to translation, including 63 low-translation-associated and 49 high-translation-associated RNA secondary structure motifs (Supplementary Table 2). In particular, we identified low-translation-associated RNA secondary structure motifs with high guanine-cytosine (GC) base pairs, such as the RNA secondary structure motif with four GC base pairs in the stem (Fig. 4b). Interestingly, we also identified high-translation-associated RNA structure motifs with a balanced ratio of GC and adenine-uracil (AU) base pairs such as the RNA structure motif with four base pairs formed by two repeats of adenine-cytosine-guanine-uracil (ACGU) (Fig. 4c).

To validate our identified RNA secondary structure motifs important to translation, we conducted experimental validation using the dual-luciferase reporter assay in plants^12^. For the high-translation-associated RNA secondary structure motif with four base pairs formed by two repeats of ACGU, we changed the two AU base pairs to the two GC base pairs, resulting in a significant decrease in TE with a reduction of up to 5.3-fold (Fig. 4d). By contrast, when we exchanged the low-translation-associated RNA secondary structure motif with four GC base pairs in the stem for the high-translation-associated RNA secondary structure motif with a balanced mix of GC and AU base pairs, we found a significant increase in TE (Fig. 4e). In particular, when we completely disrupted this low-translation-associated RNA structure motif, resulting in complete single strandedness, we observed an even greater enhancement of TE up to 2.1-fold (Fig. 4f). Our results demonstrate that PlantRNA-FM is capable of determining functional RNA secondary structure motifs in plants.

### PlantRNA-FM identifies the functional rG4s

rG4s are one of the RNA tertiary structure motifs formed by the stacking of two or more G-quartets, composed of four guanines held together by both Watson–Crick and Hoogsteen hydrogen bonds^8,33,34^. Previous studies have demonstrated the important role of individual rG4s in repressing translation^35^. However, it is impossible to identify all the rG4 motifs important to translation from tens of thousands of rG4 motifs across the transcriptome. Therefore, we took advantage of our PlantRNA-FM to identify the translation-associated rG4s at the transcriptome-wide scale.

We first obtained all the rG4 motifs in the 5′ UTRs from our G4Atlas database^34^. Subsequently, we identified all the rG4 motifs associated with translation using our model’s attention contrast matrix across the transcriptome (Methods). In particular, we only identified rG4 motifs associated with low TE, particularly with both GGA and GGU repeats (Supplementary Table 3). Therefore, our results indicate that rG4 serves as a translation repressor, which agrees with previous studies on individual rG4s^36–38^. To validate our identified translation-associated rG4 motifs, we conducted the experimental validation using a dual-luciferase reporter assay in plants^12^. We fused the 5′ UTRs containing our identified rG4 motif and the corresponding disrupted rG4 motif with the luciferase reporter genes^12^. We then measured the corresponding TEs in plants and observed a significant increase of up to 5.8-fold in the disrupted rG4 motif compared with the TE in the native rG4 motif (Fig. 4g). These results indicate that our PlantRNA-FM is also capable of identifying functional RNA tertiary structure motifs such as translation-associated rG4 motifs throughout the transcriptome.

## Discussion

In this study, we developed PlantRNA-FM, a high-performance and interpretable plant-specific RNA FM. PlantRNA-FM (Fig. 1) is designed to understand RNA sequence and structure information rather than generation. This state-of-the-art model was specifically designed based on the extensive plant RNA information from 1,124 plant species, thereby capturing the remarkable diversity of plant RNA features. From the perspective of the dataset, we have incorporated RNA sequence information of all the RNAs from the transcriptomes across 1,124 plant species. We also incorporated the corresponding RNA annotation information. The integration of RNA structure information in our PlantRNA-FM achieves superior performance in RNA structure prediction tasks compared with other FMs (Extended Data Fig. 1). Regarding the model architecture, we adopted a fine-grained tokenization method with single-nucleotide resolution. This contrasts with commonly used tokenization methods, such as byte pair encoding and *k*-mers, which rely on frequency-based tokenization and may inadvertently fragment the RNA structure motifs into arbitrary pieces. This strategy ensures the precise extraction and preservation of RNA structure motifs as coherent units throughout the pretraining process, thereby maintaining the integrity of crucial structure information. Additionally, PlantRNA-FM integrates rotary position embedding, a technique that has proven effective in enhancing the modelling capabilities for long tokens in large FMs^39^. The implementation of rotary position embedding leads to an approximately 30% reduction in the number of parameters in the embedding layer, consequently improving the efficiency of RNA tokenization and modelling.

The superior performance of PlantRNA-FM can be further demonstrated in the plant-specific downstream tasks (Fig. 2a). Our PlantRNA-FM achieved the best F1 scores of 0.974 and 0.958 for the genic region annotation in *Arabidopsis* and rice, whereas our PlantRNA-FM also achieved much better performance in predicting TE compared with other FMs (Fig. 2b,c). The outperformance of our PlantRNA-FM is probably due to the combination of both RNA sequence and structure information in our pretraining dataset, highlighting the importance of RNA structure—a key RNA feature—in regulating RNA biological processes.

In particular, we developed an interpretable framework for our PlantRNA-FM to explore the RNA features within the 5′ UTR that influences translation (Fig. 3a). Using the attention contrast matrices, we found that the nucleotides in the regions close to the start codon affect the translation the most, emphasizing the importance of positional information of functional RNA motifs (Fig. 3b). In contrast to conventional meta-gene analysis, our PlantRNA-FM is capable of providing the positional information of RNA motifs across transcriptomes, which is critical for biological regulatory functions. Furthermore, the Kozak sequence, an evolutionary conserved translation-associated sequence motif across translation initiation sites was successfully identified in both *Arabidopsis* and rice using our PlantRNA-FM (Fig. 3c,d). This result successfully demonstrates the capability of our PlantRNA-FM in identifying the RNA sequence motifs important to translation across the transcriptomes. By using an unsupervised hierarchical clustering strategy to explore our attention contrast matrix, we further systematically identified RNA secondary and tertiary structure motifs that are functionally important to translation (Fig. 4a). In particular, we identified both high-translation-associated and low-translation-associated RNA secondary structure motifs in which their differences are mainly in the strengths of the base pairs (Fig. 4b-c). This suggests that RNAs may adopt different RNA structure motifs with diverse folding strengths in regulating biological processes such as translation.

In contrast to conventional meta-gene analysis, our PlantRNA-FM is capable of delivering a comprehensive understanding of functional RNA motifs such as the type of RNA motifs, the genic position of the RNA motifs, the positive or negative effects of the RNA motifs on their functions and the exact contributions of the RNA motifs to their functions. For instance, the high GC content in 5′ UTR is anti-correlated with TE^40–42^. However, these correlations cannot facilitate the understanding of which type of regulatory motifs with high GC content repress translation. Here our PlantRNA-FM revealed diverse RNA structure motifs such as the RNA secondary structure motif with four GC base pairs in the stem and rG4s, serving as low-translation-associated RNA motifs. This suggests the diversity of RNA regulatory motifs across the transcriptomes (Fig. 4b).

## Conclusion

In summary, we have built the first interpretable RNA FM with both RNA sequence and structure information. Our PlantRNA-FM was pretrained using 1,124 plant transcriptomes. We have demonstrated that our PlantRNA-FM is capable of identifying functional RNA motifs such as translation-associated sequence and structure motifs across the transcriptomes. Through our experimental validations, we have elucidated novel translation-associated RNA motifs in plants. Our FM can be extended to explore functional RNA motifs in other kingdoms and investigate RNA motifs important for other biological functions such as RNA decay and maturation. Our PlantRNA-FM is poised to transform the way we determine RNA motifs for regulating gene expression, opening new horizons for programming RNA codes to facilitate crop improvements and RNA-based applications.

## Methods

### Pretraining datasets curation

The plant transcriptome data used for pretraining PlantRNA-FM were obtained from the 1KP dataset^14^. Note that modelling genomic sequences differs from natural language modelling. For instance, although RNA sequences are one dimensional, they strictly follow biological genomic patterns and depend heavily on certain structural characteristics. By contrast, natural language models are more resilient and can tolerate linguistic errors such as typos and grammatical mistakes. Therefore, it is important to conduct a dedicated RNA sequence curation to minimize the impact of noisy data and enhance the modelling performance. Specifically, our data curation protocol is as follows.

- **Sequence truncation and filtering**: we utilized de novo assembled transcripts and protein annotation files within the 1KP dataset, which allowed us to differentiate between 5′ UTR, CDS and 3′ UTR regions^14^. During data processing, RNAs without protein annotations were excluded. Next, we truncated RNA sequences exceeding 1,026 nucleotides to comply with the model’s maximum length capacity and filtered out sequences shorter than 20 nucleotides to eliminate noise, such as RNA fragment sequences. To address sequence similarity concerns, we used CD-HIT-EST (cd-hit-est -c 0.8) to remove sequences with an 80% sequence identity cut-off^43–45^.
- **RNA secondary structure annotation:** given the important impact of RNA secondary structures on sequence function, we annotated the local RNA structures of all the RNA sequences using ViennaRNA (with parameters maxBPspan = 30)^26^.

### RNA tokenization

We leveraged the single-nucleotide tokenization method within PlantRNA-FM because single-nucleotide tokenization can provide fine-grained, base-level interaction sequence modelling. Note that this tokenization method differs from the byte pair encoding and *k*-mers, which tokenize RNA sequences into tokens containing coupled bases. Note that these coupled bases will lead to granularity problems in downstream tasks like secondary structure prediction. This is because the structural labels correspond to single-nucleotide bases, whereas the fine-grained information may suffer losses from the features of coarse-grained token representations. In our vocabulary, we consider four types of effective base, that is, {A, T, C and G}. Similar to other encoder-only models like BERT, we incorporated special tokens (for example, <mask>, <cls>, <pad>, <eos> and <unk>) to enable masked language modelling (MLM). Furthermore, to enhance the generalization capability of PlantRNA-FM, particularly for applications involving special nucleotide bases, we have prepared placeholder tokens in the vocabulary to allow customizing the base inputs. Note that these modifications are designed to enable the strong flexibility of PlantRNA-FM for potential future applications, whereas they have not been utilized in this study. Last, we applied the rotary position embedding in the positional embedding in PlantRNA-FM, given that it supports variable sequence lengths compared with absolute position embedding.

### Model architecture

In this study, we developed PlantRNA-FM, a specialized language model based on the transformer architecture (Fig. 1). PlantRNA-FM has 35 million parameters, including 12 transformer network layers, 24 attention heads and an embedding dimension of 480. We applied layer normalization and residual connections both before and after the encoder block. As our focus is on understanding RNA rather than generating it, we only utilized the encoder component of the transformer architecture. Although the context length is set as 512 during the pretraining stage, we set the maximal modelling length to 1,026 tokens in fine tuning. This setting makes it compatible with consumer-grade GPUs, such as the NVIDIA RTX 4090, with a batch size of 16. The model was trained on four A100 GPUs over a period of three weeks, completing three epochs.

### Pretraining strategies of PlantRNA-FM

To develop an RNA FM for exploiting all the potential patterns within RNA sequences, we investigated the biological domain knowledge of RNA sequences and proposed three self-supervised pretraining objectives to enhance the foundational model.

#### Pretraining with masked nucleotides modelling

Inspired by the concept of MLM in NLP, we introduced MNM for RNA sequences. This approach involves randomly masking a portion of nucleotides and leveraging the model itself to reconstruct these masked nucleotides. Note that the ability to accurately reconstruct masked nucleotides indicates that the model is empowered with the capability of understanding RNA sequence. MNM dynamically selects 20% of the nucleotides for masking in each input sequence, as opposed to the fixed 15% masking used in the classic MLM objective designed for shorter natural language sentences. This increased masking ratio is chosen to enhance MNM’s modelling capability, considering that RNA sequences typically contain around 1,000 bases. Specifically, 10% are replaced with a <mask> token, 5% with random nucleotides and the remaining 5% are left as is. This approach, which aims for token classification, uses cross-entropy as the loss function to enhance the model’s predictive accuracy for masked or replaced nucleotides. The loss function *L*_MLM_(*θ*) for MLM is defined as follows:

$${L}_{{\rm{MLM}}}(\theta )=-\frac{1}{|m|}\sum _{i\in m}\log \left[{p}_{\theta }({x}_{i}| x\backslash i)\right],$$

where *θ* and *m* are the parameters set inside the FM and the number of masked nucleotides, respectively. *p*_*θ*_(*x*_*i*_|*x*\*i*) indicates the probability of predicting the masked nucleotide *x*_*i*_ based on its context (*x*\*i*).

#### Pretraining with RNA structure prediction

We hypothesize that effectively aligning RNA sequences with their corresponding secondary structures is important during the pretraining phase. In practice, we annotated the secondary structures within the 1KP dataset, which comprises 50 billion nucleotides. This establishes a robust foundation for our model to recognize the critical role of secondary structures. On the basis of these annotated data, we utilized cross-entropy as the loss function to predict the RNA secondary structure:

$${L}_{{\rm{SSP}}}(\theta )=-\mathop{\sum }\limits_{i=1}^{N}\mathop{\sum }\limits_{c=1}^{C}{y}_{i,c}\,\log \left[p(\;{y}_{i,c}|x;\theta )\right],$$

where *N* is the length of the RNA sequence, that is, the total number of nucleotides in the sequence; *C* denotes the number of predictions for each nucleotide (for example, ‘(’, ‘)’, ‘.’); *y*_*i*,*c*_ is the prediction of the *i*th nucleotide *c*; and *p*(*y*_*i*,*c*_|*x*; *θ*) is the probability predicted by the model parameterized by *θ*. *L*_SSP_(*θ*) is the loss function that quantifies the discrepancy between the model’s predicted probabilities for each nucleotide’s secondary structure and the actual structure, with the aim of minimizing this loss to improve the model’s accuracy in the secondary structure prediction.

#### Pretraining with RNA annotation prediction

RNA sequences exhibit notable variation across different regions, each serving distinct functions within an organism. Beyond the two aforementioned training objectives, the third one focuses on classifying regions within RNA sequences. The loss function is as follows:

$${L}_{{{\rm{CLS}}}}\left(\theta \right)=-\mathop{\sum }\limits_{i=1}^{N}\mathop{\sum }\limits_{r=1}^{R}{y}_{i,r}\log \left[ p\left({y}_{i,r}|x;\theta \right)\right],$$

where *N* is the length of the RNA sequence, that is, the total number of nucleotides or segments considered for classification. *R* represents the number of region categories we are classifying, including CDS, 3′ UTR and 5′ UTR. *y*_*i*,*r*_ is the prediction of the *i*th nucleotide *r*. *p*(*y*_*i*,*c*_|*x*; *θ*) is the probability predicted by the model, with parameters *θ*, for the *i*th nucleotide given the RNA sequence *x*. *L*_CLS_(*θ*) is the cross-entropy loss function aimed at training the model to identify different regions.

### Fine tuning of downstream tasks

The fine-tuning phase consists of three steps. First, we gathered an annotated dataset specific to each downstream task, which consists of sequences and their corresponding labels. Note that we presliced any sequences that exceed the model’s maximum length to ensure compatibility. In addition, we filtered out sequences with greater than 80% sequence identity by CD-HIT-EST (cd-hit-est -c 0.8) to prevent potential data leakage during the train-test dataset split^43^. Next, using the pretrained FM as a starting point, we adapted the output layer to accommodate the requirements of RNA modelling tasks, which may include outputting sequences, labels or scalar values. Finally, the training and inference processes are tailored to the demands of each downstream task by selecting task-specific optimizers, loss functions and tuning hyperparameters to achieve optimal performance. The source code for our training and inference can be found in our Huggingface repository^46^.

### RNA genetic region annotation and RNA structure data processing

The 5′ UTR, CDS and 3′ UTR annotations for *A. thaliana* and *O. sativa* were obtained from Phytozome v. 13 using Oryza sativa v. 7.0 and TAIR10, respectively^47^. RNA sequences shorter than 50 nucleotides were excluded. The RNA structure data used for fine tuning were derived from the TR0-TS0 dataset of bpRNA, the ArchiveII dataset and the RNAStralign dataset^27–29^. Before fine tuning, these datasets were preprocessed to a maximal length of 1,026 nucleotides and subjected to CD-HIT-EST to reduce sequence redundancy.

#### Polysome profiling mapping and data processing

Raw polysome profiling sequencing data for *A. thaliana* were obtained from published research^12^. For rice, we performed polysome-seq using the same protocol as *Arabidopsis*^8^. The genomes and annotation files of *O. sativa* and *A. thaliana* were obtained from Phytozome v. 13 with versions of Oryza sativa v. 7.0 and TAIR10 (ref. ^47^). After extracting the transcriptome sequence through the reference genome and annotation files, clean polysome profiling and RNA sequencing reads were mapped to the reference transcriptome using HISAT2 and followed by library normalization and quantification using DESeq2 (refs. ^48–50^). Next, genes with a reads per kilobase per million mapped reads of less than 1 were removed, and the TE of each gene was calculated by dividing the polysome-associated RNA levels (polysome profiling RNA sequencing) by the corresponding RNA levels (RNA sequencing)^12^. Subsequently, the dataset was classified as high or low TE, using the mean ± standard deviation threshold, and were assigned the labels 1 and 0 for high and low TE, respectively.

### RNA structure motif identification approach

#### Extraction of the attention contrast matrix

To facilitate better model interpretation, we created two additional models. One is the true model, denoted as PlantRNA-FM(+), trained using real TE labels, whereas the other one is the background model, PlantRNA-FM(–), altered using the same dataset but with randomly assigned labels. Specifically, we fine-tuned the pretrained PlantRNA-FM(+) and PlantRNA-FM(–) on each dataset for 100 epochs, using regular hyperparameter settings. To avoid overfitting, we used an early stopping strategy to terminate the fine-tuning process when the best F1 score remained unchanged for 30 epochs. Once the fine tuning was completed, we used the fine-tuned models to predict each dataset and derive the raw attention score matrices corresponding to each RNA sequence. Since the raw attention score matrices are five dimensional, we reshaped them through average-based downsampling to generate attention contrast matrices. Finally, we subtracted the attention contrast matrices of PlantRNA-FM(+) from those of PlantRNA-FM(–). Furthermore, we padded any negative values in the attention contrast matrices with zeros. The *z* scores were calculated to determine the high attention contrast. The process began with the computation of mean and standard deviation of the attention values across all the RNA sequences. Following this, the *z* score for each nucleotide position was calculated. A *z*-score threshold of 2.576 was utilized to identify nucleotides with significantly high attention values, corresponding to a 1% significance level (two tailed) in statistical hypothesis testing.

#### Generation of the RNA structure motif seed library

To identify RNA structure motifs, we first generate a library that contains RNA structure motif seeds derived from RNA sequences across the transcriptomes. In this work, we apply the Zuker algorithm from the ViennaRNA package to obtain all the suboptimal RNA structure foldings for each RNA in our dataset^35,36^. We restrict the length of the RNA structure motifs to a maximum of 30 (ref. ^51^). The folded RNA structures are then annotated using bpRNA. Subsequently, all the RNA structure motifs are extracted to generate a seed library of RNA structure motifs for the plant transcriptomes^27^. To obtain reliable RNA structure motifs, we set the range of RNA structure stems from 4 to 7, and the loop length from 4 to 9.

#### Identification of translation-associated RNA secondary structure motifs

From the previous step, we obtained all the potential foldings of the RNA structure motif in the 5′ UTR and aligned them with the attention contrast matrix. We evaluated each RNA structure motif using a paired *t*-test to obtain a *P* value. Then, we corrected the obtained *P* value using the Benjamini–Hochberg method. RNA structure motifs with *P* values of less than 0.01 were considered significant and extracted as the high attention RNA structure motifs. Then, we extracted their corresponding RNA sequence and converted them into numerical matrices using the one-hot encoding method. Subsequently, we applied an unsupervised hierarchical clustering strategy to classify the nucleotides corresponding to the positions of the RNA structure pairs into 2–100 clusters^52^. For each cluster containing a minimum of 30, the significance was assessed using Fisher’s exact test for high attention RNA structure motifs. RNA motifs with an odds ratio over 1 and a *P* value below 0.05 were identified as high-translation-associated motifs. On the contrary, those with an odds ratio of less than 1 and a *P* value below 0.05 were associated with low TE. Additionally, we calculated the mean information content of all the bases, defined as the average positional information content. RNA motifs with an average positional information content below 1.5 were excluded from further analysis.

#### Identification of translation-associated rG4s

We obtained all the potential rG4s in rice from our G4Atlas database^34^. Next, we aligned the rG4 sequences with the corresponding attention contrast matrix and used the paired *t*-test to assess the statistical significance. For each length of rG4, we adjusted its *P* value using the Benjamini–Hochberg correction method and selected rG4s with a *P* value of less than 0.01 as the high attention rG4s.

### Plasmid construction and dual-luciferase reporter assay

The 5′ UTR sequences of LOC_Os12g19470.2, LOC_Os12g02250.1 and LOC_Os08g40620.1 were synthesized and incorporated into inter2 immediately upstream of the ATG of the firefly luciferase gene by Universe Gene Technology. Dual-luciferase reporter assay was conducted as previously described^12^. Briefly, sequencing-validated vectors were transformed to *Agrobacterium tumefaciens* GV3101 and infiltrated into four-week-old tobacco *Nicotiana benthamiana* leaves. Here 10 mg of leaf discs were harvested and ground into a fine powder in liquid nitrogen after 72 h and homogenization in passive lysis buffer (Promega). After centrifugation, the clear supernatant was diluted 20-fold with a passive lysis buffer and subjected to dual-luciferase assay using the Dual-Luciferase Reporter Assay System (Promega). Quantitative polymerase chain reaction was performed to determine the levels of firefly luciferase. Protein level or mRNA abundance of firefly luciferase was normalized to that of internal control *Renilla* luciferase. The normalized firefly luciferase protein level was adjusted in relation to the normalized mRNA abundance to calculate the raw TE. The relative TE was subsequently computed by normalizing it to the raw TE of the wild type. Primers used are listed in Supplementary Table 4.

## Supplementary information


**Supplementary Table 1** Comparison of F1 scores of different pretrained models on the RNA structure prediction task. **Supplementary Table 2** Identified translation-associated RNA structure motifs. *P* value was calculated using a two-sided Fisher’s exact test. **Supplementary Table 3** Identified translation-associated rG4. *P* value was calculated using a two-sided Fisher’s exact test. **Supplementary Table 4** Primers used for quantitative reverse-transcription polymerase chain reaction.


## Source data


Source Data Fig. 2Statistical source data.
Source Data Fig. 3Statistical source data.
Source Data Fig. 4Statistical source data.
Source Data Extended Data Fig. 1Statistical source data.


## Data Availability

The polysome-seq sequence data of *A. thaliana* was obtained from the Sequence Read Archive (SRA) (https://www.ncbi.nlm.nih.gov/sra) under BioProject ID number PRJNA762705 (ref. ^8^). The raw sequence data of *O. sativa* has been deposited in the SRA under BioProject ID number PRJNA1112739. Source data are provided with this paper.

## Code availability

The source code of this study is freely available via Huggingface at https://huggingface.co/yangheng/PlantRNA-FM (ref. ^46^).
